# No Evidence for Enrichment in Schizophrenia for Common Allelic Associations at Imprinted Loci

**DOI:** 10.1371/journal.pone.0144172

**Published:** 2015-12-03

**Authors:** Valentina Escott-Price, George Kirov, Elliott Rees, Anthony R. Isles, Michael J. Owen, Michael C. O’Donovan

**Affiliations:** MRC Centre for Neuropsychiatric Genetics & Genomics, Cardiff University, Cardiff, United Kingdom; Johns Hopkins Bloomberg School of Public Health, UNITED STATES

## Abstract

Most genetic studies assume that the function of a genetic variant is independent of the parent from which it is inherited, but this is not always true. The best known example of parent-of-origin effects arises with respect to alleles at imprinted loci. In classical imprinting, characteristically, either the maternal or paternal copy is expressed, but not both. Only alleles present in one of the parental copies of the gene, the expressed copy, is likely to contribute to disease. It has been postulated that imprinting is important in central nervous system development, and that consequently, imprinted loci may be involved in schizophrenia. If this is true, allowing for parent-of-origin effects might be important in genetic studies of schizophrenia. Here, we use genome-wide association data from one of the world’s largest samples (N = 695) of parent schizophrenia-offspring trios to test for parent-of-origin effects. To maximise power, we restricted our analyses to test two main hypotheses. If imprinting plays a disproportionate role in schizophrenia susceptibility, we postulated a) that alleles showing robust evidence for association to schizophrenia from previous genome-wide association studies should be enriched for parent-of-origin effects and b) that genes at loci imprinted in humans or mice should be enriched both for genome-wide significant associations, and in our sample, for parent-of-origin effects. Neither prediction was supported in the present study. We have shown, that it is unlikely that parent-of-origin effects or imprinting play particularly important roles in schizophrenia, although our findings do not exclude such effects at specific loci nor do they exclude such effects among rare alleles.

## Introduction

Schizophrenia is highly heritable disorder [1, 2] for which there is evidence for a substantial neurodevelopmental component [3–5]. Risk of schizophrenia is conferred by a large number of genetic factors; genome-wide association studies (GWAS) have identified over 100 schizophrenia-associated common variant loci [6] as well as 11 robustly associated rare chromosomal copy number variants (CNVs) [7]. How these co-act with environmental exposures, which also influence risk, is as yet unknown.

Imprinting describes the phenomenon whereby genes are subject to regulation by an epigenetic mechanism such that gene expression depends on whether the gene is inherited from the mother or the father. Genes that are known to be imprinted are enriched for expression in the placenta and the brain [8]. It has been postulated that imprinted genes may be important contributors to complex trait variation [9, 10] in general and psychiatric disorders in particular [11]. If this is true, then at the relevant loci, only alleles present in one of the parental copies of the gene, the expressed copy, is likely to contribute to disease (unless a genetic variant causes activation of a usually silenced copy).

There is already one example of a known imprinted schizophrenia risk locus, a duplication CNV at 15q11-q13 [7, 12]. At this locus, only maternally inherited duplications are pathogenic, leading to the hypothesis that the causal factor is overexpression of the maternally expressed imprinted gene *UBE3A* [7, 12], at least for psychotic illness. Although imprinting is one of most well-known parent-of-origin effects, there are other mechanisms that may lead to modification of the phenotype through parent-of-origin effects [13], for example biases in *de novo* mutational rates.

Studies of schizophrenia and other complex traits are generally based on case-control samples. In these samples, it is not possible to determine the parental origin of autosomal alleles, except where this can be inferred by assaying the epigenetic status of the imprinted cluster [7, 12]. Aiming to determine whether there is any evidence to support the hypothesis that imprinted loci are relatively enriched in schizophrenia [11], we have conducted parent-of-origin analyses for 695 complete parent-proband trios. The sample, though large for a sample of trios, is small relative to GWAS case-control samples and is therefore weakly powered to detect individual loci at genome wide levels of significance. Therefore instead taking a genome-wide perspective, we adopted a hypothesis based approach in which we constrained the analyses to loci that we postulated would be most likely to be enriched for schizophrenia relevant imprinted genes. We first tested the hypotheses that alleles with robust prior evidence for association to schizophrenia are enriched for parent-of-origin biased transmission in our trios, and that the loci defined by the associated alleles are enriched for imprinted genes. In a complementary analysis, we tested the inverse of the above hypothesis, namely, that loci thought to be imprinted are enriched for association signals in the large recently published GWAS dataset of the Psychiatric Genomics consortium [6], and for parent-of-origin effects in the trios.

## Materials and Methods

### Parent-Proband Trios data

For the primary parent-of-origin analysis we used a dataset comprising 695 parent-offspring trios from Bulgaria typed with the Affymetrix GeneChip^®^ 6.0 Array as previously described [14, 15]. Families with more than 3% Mendelian errors (considering all SNPs) were discarded. The final average Mendelian error rate was 0.09% [SD = 0.2%; maximum 2.98%]. We included only autosomal markers with minor allele frequencies ≥1% and with Hardy–Weinberg Equilibrium p-values ≥10^−6^ in parents. Markers with Mendelian errors >1% were excluded. We did not use imputed data since array genotypes provide good coverage of the main loci of interest and moreover, the optimal analytic methods for parent-of-origin require genotypes rather than genotype probabilities.

### Schizophrenia Associated Loci

We defined schizophrenia associated variants as those from independent loci reported to be genome-wide significant in the recent publication from the Schizophrenia Working Group of the Psychiatric Genomics Consortium [6] (PGC). That study reported at total of 108 independently associated loci, of which 105 were autosomal. Other schizophrenia association data were obtained as summary statistics from the ricopili website that the PGC has provided for public access (http://www.broadinstitute.org/mpg/ricopili/). Although most of the present sample was included in that study, parent-of-origin effects were not evaluated. Our present analysis for those effects is therefore independent of any previously reported data.

To test whether GWAS significant associated markers are enriched for parent-of-origin effects, we used a) one of the index markers for each locus or a perfect proxy for it or where none was available, either b) the best proxy (r^2^≥0.8) for it or c) another GWAS significant marker at the locus. To test whether the loci are enriched for imprinted genes in mice and humans, we used the genes (N = 350) that are present in the physical regions from Extended Data of primary GWAS manuscript of the Schizophrenia Working Group of the PGC [6]. The MHC region is within a very large region of LD (8 million bases or so) spanning hundreds of genes. As any one of hundreds of genes might be responsible, the addition of this locus to gene set analysis compromises power by introducing 100s of non-associated genes into the analysis merely to capture a single association. Thus genes mapping to the MHC region were excluded.

### Imprinted genes in human and mice

We identified from the GeneImprint database (www.geneimprint.com) a total of 197 genes reported as imprinted in humans (N = 177) and/or mice (N = 93, in mice only N = 20). We identified 150 genes reported as robustly imprinted in mice from the MouseBook database (www.mousebook.org). Mouse genes were converted to their human orthologues using the conversion tool from the MGI Biomart database (biomart.informatics.jax.org). In total, we identified 247 unique autosomal genes of which 225 contained at least one marker in the PGC schizophrenia dataset (S1 Table). Markers were assigned to genes if they were located within the genomic sequence lying between the start of the first and the end of the last exon of any transcript corresponding to that gene. The chromosome and location for all currently known human SNPs were taken from the dbSNP132 database, as was their assignment to genes (using UCSC hg19/NCBI build 37).

### Statistical analysis

Single marker parent-of-origin p-values were calculated using a log-linear approach as implemented in UNPHASED[16]. The log-linear approach is designed to analyse parent-proband trio data based on the maximum likelihood ratio test comparing two models with and without stratification on parental mating type. This method yields a likelihood ratio test (an alternative to Transmission Disequilibrium Test (TDT) [17]) in the simple situation when measuring the over-transmission of an allele from heterozygous parents to affected offspring, and is easily extended to include tests for parent-of-origin. It has been shown to have greater power than TDT-inspired tests of parent-of-origin [18].

To assess for enrichment of parent-of-origin effects among genome-wide significant markers (or their proxies) reported by the PGC [6] we used an exact binomial test. This compares the number of nominally significant (p≤0.05) parent-of-origin association p-values in the PGC-derived set to the expected number of nominally significant p-values in a random set. Note that the associated markers are already pre-defined as indexing independent associations, requiring no further adjustments for potential non-independence of the tests.

To test for enrichment of significant associations in the genes known to be imprinted in humans and/or mice in the PGC dataset, we performed a bootstrapping process in which we randomly selected the same number of genomic regions from that dataset as there are putatively imprinted genes, ensuring each locus contained the same number of SNPs as did its matched imprinted gene. We then compared the number of significant SNPs in the imprinted genes with the number of significant SNPs in the 1000 times randomly bootstrapped regions.

## Results

Genome-wide, there was no evidence for inflation in parent-of-origin test statistics (S1 Fig). For 63 of the index markers at 105 autosomal GWS loci, we had in the Bulgarian trios, either a directly genotyped marker or a perfect proxy (r^2^ = 1) of it (S2 Table). Three of these had nominally significant parent-of-origin effect (minimal p = 0.025, S2 Table). The enrichment p-value was 0.616 (one-sided exact binomial test). Proxies with r^2^ greater than 0.8 but smaller than 1 were available for 21 SNPs, only one of which had a nominally significant parent-of-origin effect (p = 0.022, S2 Table). At an additional 4 loci, we had a genotyped marker that while in modest LD (r^2^<0.8) with an index marker, was genome-wide significant in the PGC data, of which 1 had a nominally significant parent-of-origin effect (P = 0.028). Thus, in total, of 88 markers representing 105 genome-wide significant loci, 5 had nominally significant parent-of-origin effects which is not more than expected by chance (p-value = 0.451).

In the PGC dataset, autosomal schizophrenia-associated loci span 350 genes [6]. Of these only one (*ACD* (chr16:67,691,415–67,694,718)) was also on the list of 225 genes which are reported as possibly imprinted in humans or mice (S1 Table). However, our proxy (r^2^ = 0.74) for the index SNP that is associated with schizophrenia in the PGC shows no significant parent-of-origin effect in the trios (p = 0.875, S2 Table). The observed overlap between schizophrenia-associated genes and imprinted genes was not significant (p = 0.53, Fisher’s exact test). The mean number of markers per imprinted gene was ~303 (SE = 45.5, median 73). Given that schizophrenia associated genes have a greater number of markers (mean ~347, SE = 69.4, median = 82), than is typical for all genes (mean ~143 markers, SE = 2.4, median 32), the fact that imprinted genes are similarly enriched for those with larger numbers of markers should create a bias whereby imprinted genes have a better than average chance (by virtue of multiple testing) of being associated with schizophrenia. That we do not see this suggests there is no general enrichment of imprinted genes among those conferring risk of schizophrenia.

There were 4,832 SNPs in the parent-proband trios data mapping to the 225 putative imprinted genes which we gathered from public databases. The most significant parent-of-origin effect (rs4758621, p = 8.2x10^−5^, chr11:3,009,640, *NAP1L4* gene) was not significant after correction for multiple testing (empirical p-value = 0.339 estimated using the bootstrapping procedure). We also did not find evidence for significant enrichment of parent-of-origin effects among the markers mapping to imprinted genes at any of the significance thresholds we examined (Table 1). We undertook the same analysis restricted to the 177 genes annotated as imprinted in humans and obtained similar results (S3 Table).

**Table 1:** Test for overrepresentation of parent-of-origin effects (top section) and markers associated with schizophrenia (bottom section) in genes imprinted in humans or mice.

P(T)	N of observed significant SNPs	Expected number of significant SNPs (mean [SD])	Overrepresentation p-value
Trios:			
0.001	9	6.0 [4.16]	0.312
0.01	45	52.1 [12.9]	0.638
0.05	233	247.7 [31.7]	0.618
PGC SZ:			
0.001	572	828.9 [338.5]	0.772
0.01	2,397	2671.9 [586.0]	0.664
0.05	6,750	7131.0 [835.4]	0.648

Legend: P(T) denotes the significance threshold for association surpassed by the numbers of markers denoted in the same row. Third column shows the number of observed significant SNPs in the parent-proband trios and PGC SZ data, respectively. The expected numbers of markers (mean) and standard deviations (SDs) were estimated using 1000 simulations by randomly bootstrapping the same number of loci, each with the corresponding numbers of markers, as the set of genes imprinted in humans or mice. Over-representation p-values were estimated empirically as the proportion of simulations where the number of significant markers was greater or equal to that observed in real dataset.

In the PGC schizophrenia data, the 225 genes imprinted in humans and/or mice were spanned by 67,883 markers. To derive an approximation for experiment-wide significance level in this set of markers, we used bootstrapping. At each of 1000 simulations we sorted 67,883 p-values in ascending order and recorded the 3395th smallest p-value (corresponding to a null P of 0.05). The average of all such recorded p-values across the 1000 simulations (p≤1.97x10^−6^) was taken to be the experiment-wide significance level. A total of 35 markers spanning six distinct genes surpassed this threshold; markers at *QPCT* (chr2:37,571KB-37,600KB), *BTNL2* (chr6:32,362KB-32,375KB), *LIN28B* (chr6:105,443KB -105,472KB), *MAGI2* (chr7:77,646KB-79,083KB), *GLIS3* (chr9:3,824KB-4,300KB) and *NTM* (chr11:131,240KB-132,207KB).

After removing markers in LD (r^2^>0.5) with the most strongly associated PGC marker in each gene, we retained 6 independent SNPs, one per gene (Table 2). In this table we also show the best proxies within 500KB available in the parent-proband trios data. For 4 of these 6 SNPs we had good proxies (r^2^≥0.8). On chromosome 6, the SNP rs1150753 was genome-wide significant in the PGC schizophrenia data (OR = 1.167 with respect to allele A, p = 2.02x10^−17^). This marker also had a significant parent-of-origin effect in the parent-proband trios data (p = 0.0019) with the schizophrenia-risk allele OR for paternally transmission being 3.16 relative to maternally transmitted alleles (i.e. the risk allele A is under-transmitted in female and over-transmitted in males). While significant in the context of 6 independent SNPs (corrected p = 0.012), this finding is very modest in the context of the multiple tests outlined above. No other marker had a nominally significant parent-of-origin effect (Table 2).

**Table 2:** PGC SZ experiment-wide significant LD-pruned (r2>0.5) SNPs in the 225 genes imprinted in humans and/or mice and their proxies in the trios data with the parent-of-origin effect (POE) p-values and PGC p-values.

Experiment-wide significant SNPs in PGC SZ					Proxy SNPs						
CHR	SNP	BP	PGC p-value	gene	SNP	BP	PGC OR	PGC p-value	POE OR	POE p-value	r^2^
2	rs3770752	37576136	2.63x10^−6^	*QPCT*	rs3770752	37576136	1.055	2.63x10^−6^	1.144	0.5381	1
6	rs147461705	32368462	8.51x10^−19^	*BTNL2*	rs1150753	32059867	1.167	2.02x10^−17^	3.158	0.0019	0.848
6	rs78370910	105472320	3.60x10^−7^	*LIN28B*	rs314272	105462004	0.948	4.6x10^−7^	1.067	0.7535	0.999
7	rs4727751	78336934	1.7x10^−6^	*MAGI2*	rs2190664	78322126	1.042	0.00012	0.803	0.3199	0.83
9	rs13301469	3911353	9.65x10^−7^	*GLIS3*	rs583963	3908216	1.054	0.00272	1.022	0.9392	0.631
11	rs35659386	131300368	7.32x10^−6^	*NTM*	rs7108020	131289820	0.983	0.1218	0.722	0.1559	0.255

Finally, in an evaluation of imprinted genes, we did not find in the PGC dataset a significant excess of associated markers surpassing a range of significance thresholds (Table 1).

## Discussion

In the present study, we have tested the hypothesis that imprinted genes are disproportionately enriched for common alleles that confer susceptibility to schizophrenia. If the hypothesis is true, we first predicted that common alleles that are associated with schizophrenia would show evidence for parent-of-origin biased transmission. However, among the index SNPs, or good proxies thereof, for 88 autosomal schizophrenia-associated loci, we found no evidence (after correction for multiple testing) that the risk associated with any individual variant was dependent on the transmitting parent-of-origin. Moreover, we did not find evidence that the set as a whole showed a larger number of nominally significant associations than expected by chance.

We additionally found no evidence to support the second prediction that known or predicted imprinted genes are enriched among schizophrenia associated loci. Indeed only 1 putatively imprinted gene (*ACD* chr16:67,691–67,695KB), mapped to such a locus (excluding genes mapping to the extended MHC region). It should be noted that *ACD* maps to an associated locus that contains at least 26 genes and therefore is by no means considered to be strongly implicated in the disorder. Moreover, we see no support for a parent-of-origin effect at this locus, suggesting the schizophrenia association is not driven by an imprinted gene.

The third prediction arising from the overarching hypothesis was that one or more parent-of-origin effects would be observed in the trios among markers spanning the 225 putatively imprinted genes. Again, we found no evidence, either at the level of any single marker or the set of markers as a whole.

Finally, aiming to constrain hypothesis testing to a minimum, in a two-step design, we aimed to combine the case-control data from the large GWAS dataset with the parent-of-origin data from the trios, restricting the analyses to putatively imprinted genes. In doing so, we identified 6 putatively imprinted genes that contained 1 or more variant that met experiment-wide significance in the case-control data, one of which (*BTNL2*) contained a variant that was actually genome-wide significant. Moreover, our best proxy for that genome-wide significant variant also showed a significant parent of origin effect in the trios. Given a previous report (in this set of trios) of a *de novo* nonsense mutation in this gene on a proband with schizophrenia [19], at face value, *BTNL2* might be considered an attractive candidate imprinted gene for schizophrenia. Additional factors, however, strongly mitigate against this conclusion. First, *BTNL2* lies within the extended MHC region of chromosome 6, a schizophrenia-associated locus that is gene dense and spans several million bases. Accordingly, the extent to which any one gene can derive candidacy from GWAS is weak. Second, the candidacy of *BTNL2* as an imprinted gene is based only on computational [20] evidence, and is as yet experimentally unconfirmed. And third, *BTNL2* is predicted to be maternally expressed (S1 Table), leading to the expectation that the risk allele should be preferentially transmitted from maternal, not as we observe, paternal chromosomes. Although, it could be an activating variant, so being preferentially transmitted from the paternal allele does not preclude it *per se*.

Our study has three important limitations. First, the databases from which we extracted imprinted genes includes not only robustly confirmed imprinted genes with known epigenetic mechanisms, but also genes predicted to be imprinted using bioinformatics techniques, and genes at which there have been observations of a parental bias in the transmission of some trait. It likely then, that this list of genes is neither comprehensive nor free of falsely annotated imprinted genes. Misclassification errors such as this will reduce power. Second, our sample of trios was large by standards of what is available world-wide, but it is not large in the context of GWAS. This means we were restrained to testing a subset of loci, albeit a subset that would most plausibly demonstrate effects if our overarching hypothesis was correct, rather than comprehensively evaluate all variants in a genome-wide context for parent-of-origin effects. Third, our focus was on common genetic variation; this study makes no evaluation of whether rare genetic risk variants might be enriched at imprinted loci.

In summary, our study suggests that imprinted genes do not play a particularly prominent role in schizophrenia. Our findings should, however, not be interpreted as meaning that imprinted genes play no role in schizophrenia, nor that parent-of-origin effects (in the statistical sense) do not occur in the disorder, although our data from this analysis are compatible with the hypothesis that they do not.

## Supporting Information

S1 Fig QQ-plot of parent-of-origin test in terms of -log_10_(p-values). The p-values were calculated with the maximum likelihood ratio test comparing two models with and without stratification on parental mating type as implemented in UNPHASED [16].
(DOCX)

S1 Table Imprinted genes in human and mice. (XLSX)

S2 Table Independent SNPs showing evidence for association in the GWAS from the Schizophrenia Working Group of the PGC with their direct matches or proxies from parent-offspring trios data. (XLSX)

S3 Table Test for overrepresentation of parent-of-origin effects (top section) and markers associated with schizophrenia (bottom section) in genes imprinted in humans. (DOCX)
