# Supplementary material for: No Evidence for Enrichment in Schizophrenia for Common Allelic Associations at Imprinted Loci
Source: PLoS One. 2015 Dec 3;10(12):e0144172. doi: 10.1371/journal.pone.0144172 (PMC4669201; doi:10.1371/journal.pone.0144172)

Supplemental Figure 1. QQ-plot of parent-of-origin test in terms of -log_10_(p-values). The p-values were calculated with the maximum likelihood ratio test comparing two models with and without stratification on parental mating type as implemented in UNPHASED^16^.


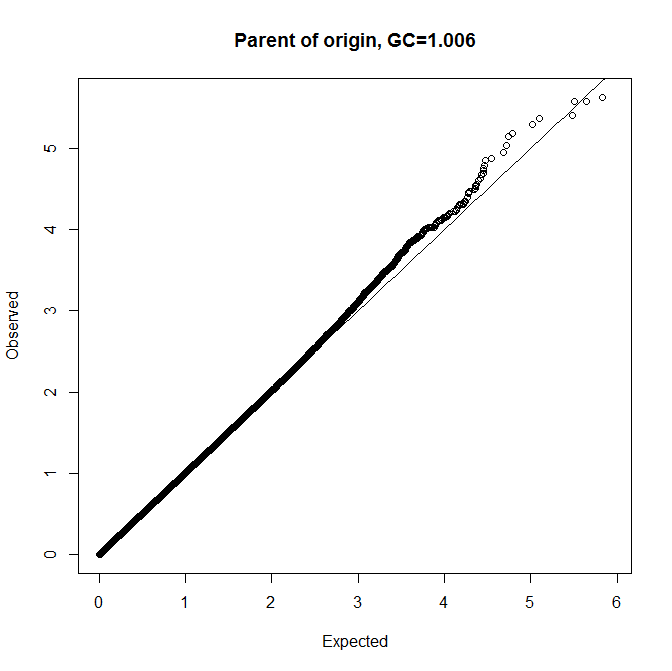

Supplement: S1 Fig — The p-values were calculated with the maximum likelihood ratio test comparing two models with and without stratification on parental mating type as implemented in UNPHASED [16]. (DOCX) [file pone.0144172.s001.docx]
