# Supplementary material for: No Evidence for Enrichment in Schizophrenia for Common Allelic Associations at Imprinted Loci
Source: PLoS One. 2015 Dec 3;10(12):e0144172. doi: 10.1371/journal.pone.0144172 (PMC4669201; doi:10.1371/journal.pone.0144172)
Supplement: S3 Table — (DOCX) [file pone.0144172.s004.docx]

**Supplementary Table 3.** Test for overrepresentation of parent-of-origin effects (top section) and markers associated with schizophrenia (bottom section) in genes imprinted in humans.

| Study | P(T) | N of observed significant SNPs | Expected number of significant SNPs (mean [SD]) | Overrepresentation p-value |
| --- | --- | --- | --- | --- |
| Trios | 0.001 | 3 | 4.4 [3.7] | 0.715 |
|  | 0.01 | 26 | 38 [11.4] | 0.624 |
|  | 0.05 | 161 | 184 [26.2] | 0.648 |
| PGC SZ | 0.001 | 532 | 521 [231.0] | 0.418 |
|  | 0.01 | 1,965 | 1,870 [443.7] | 0.374 |
|  | 0.05 | 5,186 | 5,184 [649.9] | 0.490 |

Legend: P(T) denotes the significance threshold for association surpassed by the numbers of markers denoted in the same row. Third column shows the number of observed significant SNPs in the parent-proband trios and PGC SZ data, respectively. The expected numbers of markers (mean) and standard deviations (SDs) were estimated using 1000 simulations by randomly bootstrapping the same number of loci, each with the corresponding numbers of markers, as the set of genes imprinted in humans or mice. Over-representation p-values were estimated empirically as the proportion of simulations where the number of significant markers was greater or equal to that observed in real dataset.
